# The relationships between noriuchi (stake, risk, time, outcome-sharing gambling) participation, gambling behavior, and problem gambling severity among Japanese gamblers

**DOI:** 10.3389/fpsyg.2026.1831401

**Published:** 2026-10-29

**Authors:** Goki Kohagura, Eiichi Kamimura, Kengo Yokomitsu

**Affiliations:** 1 School of Psychological Sciences, University of Human Environments, Matsuyama, Japan; 2 Institute of Humanities and Social Sciences, Niigata University, Niigata, Japan

**Keywords:** collaborative gambling, gambling frequency, Japan, noriuchi, problem gambling, stake sharing

## Abstract

**Introduction:**

In Japan, noriuchi refers to a collaborative gambling practice in which multiple players pool money used for betting and later split the final net profit or loss. This cross-sectional online study aimed (a) to estimate the prevalence of noriuchi among adult Japanese gamblers and (b) to examine how the frequency of noriuchi participation relates to gambling involvement and problem gambling severity.

**Methods:**

In February 2022, an online survey was administered via Rakuten Insight, Inc. to adults aged 20 years or older. Participants were eligible if they reported gambling at least monthly in at least one of nine gambling activities during the past 12 months (*N* = 2,000). Noriuchi participation frequency was assessed on a four-point scale (never, less than monthly, monthly or more, weekly or more). Outcomes included monthly gambling days, monthly gambling expenditure, and problem gambling severity measured with the Japanese version of the Problem Gambling Severity Index (PGSI).

**Results:**

Overall, 498 participants (24.9%) reported noriuchi experience in the past 12 months; among them, 198 (39.76%) met the PGSI criterion for problem gambling. After adjustment for demographic covariates, noriuchi participation was associated with greater problem gambling severity across several gambling activities. Associations with monthly gambling days and expenditure were more limited and varied across gambling activities, with significant increases primarily observed among those engaging in noriuchi weekly for certain gambling activities.

**Discussion:**

These findings suggest that noriuchi is associated with problem gambling severity, whereas its associations with gambling frequency and individual gambling expenditure are less consistent.

## Introduction

Gambling is commonly defined as wagering money or valuables on an activity with an uncertain outcome, such as card and dice games, electronic gaming machines, lotteries, scratch tickets, bingo, roulette, races, and sports events (Petry, 2004). In a recent meta-analysis of representative adult samples from 68 countries and regions, approximately 1.4% of adults met criteria for problem gambling in the past 12 months and 8.7% showed at-risk gambling, with higher prevalence among men (Tran et al., 2024). Problem gambling is associated not only with financial losses but also with broader harms that undermine the health and well-being of families and communities and contribute to diverse social problems (Langham et al., 2016). Accordingly, many jurisdictions have implemented harm-reduction strategies, including public education and self-exclusion programs.

Gambling engagement may be maintained by behavioral and cognitive processes such as loss chasing (Lesieur, 1979), the illusion of control over chance-determined outcomes (Langer, 1975), and cognitive biases such as the law of small numbers, whereby people overgeneralize from limited samples and place high-risk bets (Tversky and Kahneman, 1971). Beyond individual-level processes, gambling decisions are shaped by social context, including the presence of other people (Rockloff and Greer, 2011; Rockloff et al., 2012; Lemoine and Roland-Lévy, 2017).

A growing experimental literature indicates that other people can alter gambling behavior. Using an electronic gaming machine (EGM) task, Rockloff and Greer (2011) compared solitary play with play observed by a small (∼6) or large (∼26) audience and found that wager size decreased when participants were watched, particularly with larger audiences. They proposed a protective “social shield” whereby public scrutiny suppresses excessive betting. Consistently, Lemoine and Roland-Lévy (2017) observed reduced risk-taking in a roulette task when participants were observed, especially when observation included video recording. However, other studies have reported social facilitation of gambling behavior. Cole et al. (2011) found that participants gambling alongside another gambler placed more chips and made riskier bets than those gambling alone. Similarly, Hopfgartner et al. (2021), using large-scale account-based gambling data, found that gamblers staked more money and played for longer in crowded gambling environments. Taken together, the available literature suggests that the influence of social presence on gambling is complex and may either facilitate or inhibit gambling behavior depending on the social and situational context (Griffiths, 2023).

In addition, Yokomitsu et al. (2023) conducted a *post hoc* analysis among 40 moderate- to high-risk gamblers (PGSI ≥ 3) and found greater risk-taking under a cooperation condition than under a solitary condition (estimate = 1.13, SE = 0.53, *p* = 0.035). In the cooperation condition, participants pooled funds, shared a common balance, consulted each other about betting strategies and stopping rules, and divided the final outcome. In Japan, this stake-sharing practice is commonly referred to as *noriuchi*. Here, we define *noriuchi* as gambling in which multiple players share betting funds and distribute the net profit or loss among participants.

Although experimental paradigms enable precise tests of how social presence affects decision-making, the real-world prevalence of stake-sharing gambling and the extent to which its frequency is associated with gambling involvement and problem gambling severity remain poorly characterized.

Accordingly, the present study used a large-scale online survey to address questions that are difficult to examine comprehensively in laboratory settings. Specifically, using a cross-sectional sample of 2,000 Japanese adults (≥20 years) who gambled at least monthly in the past year, we (a) estimated the 12-month prevalence of *noriuchi* and (b) explored associations between *noriuchi* participation frequency and monthly gambling days, monthly gambling expenditure, and problem gambling severity.

Stake-sharing gambling may involve social and psychological processes beyond individual risk preferences or financial motives, such as diffusion of responsibility, social comparison and conformity, solidarity, and emotional sharing. Although such mechanisms may contribute to stake-sharing gambling, the present study did not directly examine these processes. Instead, we focused on the prevalence of noriuchi and its associations with gambling involvement and problem gambling severity.

## Materials and methods

### Study design and procedure

This study used a cross-sectional online survey of adults in Japan. The survey was conducted in February 2022 in cooperation with a commercial survey company (Rakuten Insight, Inc.).

The survey invitation was distributed to 210,677 members registered in Rakuten Insight’s gambling-monitor panel, and responses were collected until a final sample of 2,000 participants was obtained.

### Participants

Participants were Japanese residents aged 20 years or older who completed the online survey. At the beginning of the questionnaire, a screening section assessed past-12-month participation frequency in each of nine gambling activities using a four-point scale. Participants were included in the analytic sample if they reported engaging in at least one of the nine gambling activities ‘monthly or more’ or ‘weekly or more’ during the past 12 months. The final eligible sample comprised 2,000 respondents (1,809 men, 191 women; mean age = 51.6 years, SD = 10.08). Prior to participation, respondents were informed about the study purpose, voluntary participation, and privacy protections and were instructed to proceed with the online survey only if they agreed to participate.

### Ethics statement

To respect participants’ free will, the survey began with an explanation of the study purpose, voluntary participation, and privacy protection; only those who provided consent could proceed. The study was approved by the Ethics Committee of Kawasaki University of Medical Welfare (approval number: 21-101).

### Measures

An online questionnaire assessed demographic characteristics, noriuchi behavior, gambling involvement, and problem gambling severity. First, as a screening measure, respondents were asked whether they had engaged in any of nine types of gambling during the past 12 months. Individuals who reported participating in at least one type of gambling at a frequency of once a month or more were included in the study. Demographic variables included gender, age, occupation, educational attainment, marital status, household income, and the amount of discretionary money available per month.

#### Noriuchi experience and frequency

For each gambling activity that participants reported engaging in at least once during the previous 12 months, they were asked to report the frequency of noriuchi participation, regardless of how frequently they had engaged in that gambling activity. Therefore, participants could provide responses regarding noriuchi participation even for gambling activities in which they had participated less than once per month. They indicated the frequency with which they engaged in noriuchi in that gambling context using a four-point scale (never, less than monthly, monthly or more, and weekly or more). Participants were classified as having noriuchi experience if they selected any response other than ‘never’ for at least one applicable gambling activity; those who selected ‘never’ for all applicable gambling activities were classified as having no noriuchi experience.

#### Problem gambling severity

Problem gambling severity was assessed using the Japanese version of the Problem Gambling Severity Index (PGSI; So et al., 2019). The PGSI consists of nine items assessing gambling behavior and related problems during the past 12 months. Items are rated on a four-point scale from ‘never’ to ‘almost always’ and summed; higher total scores indicate more severe gambling-related problems. For the analyses, PGSI total scores were categorized as non-problem gambling (0), low-risk gambling (1–2), moderate-risk gambling (3–7), and problem gambling (≥8).

### Statistical analysis

All statistical analyses were conducted using R (version 4.4.3; R Foundation for Statistical Computing, Vienna, Austria). For each gambling activity, participants who had engaged in each gambling activity at least once during the previous 12 months were included in the corresponding analyses. To account for the distributional characteristics of each outcome variable, different regression models were applied. Gambling disorder severity, categorized using the Problem Gambling Severity Index (PGSI), was analyzed using ordinal logistic regression. Auto racing and casino gambling were excluded from the ordinal logistic regression analyses because sparse or zero cells in the higher-frequency noriuchi categories precluded stable model estimation. The number of gambling days per month was analyzed using negative binomial regression because of overdispersion in the count data. Monthly gambling expenditure was positively skewed and therefore log-transformed prior to analysis; associations with noriuchi participation were examined using multiple linear regression. In all regression models, noriuchi participation frequency was entered as the primary independent variable, with age, sex, marital status, and annual household income included as covariates to adjust for potential confounding. Effect estimates with 95% confidence intervals (CIs) and *p*-values are reported. Statistical significance was set at *p* ≤ 0.05.

## Results

### Participant characteristics

Among the 2,000 participants, 90.5% were men and 9.6% were women. The mean age was 51.6 years (SD = 10.08), with responses obtained across age groups. Most participants were employed, and the sample also included individuals who were unemployed as well as homemakers (Table 1). Approximately half had graduated from university, followed by those with a high school education. The median annual household income was 4,000,000 JPY (first–third quartiles: 2,000,000–6,000,000 JPY). More than half of participants were married (*n* = 1,277; 63.9%).

**Table 1:** Participant characteristics (*N* = 2,000).

	Total *n* = 2,000 *n* (%)		Noriuchi *n* = 498 *n* (%)	
Gender				
Men	1,809 (90.5)		433 (86.9)	
Women	191 (9.6)		65 (13.1)	
Age				
Means (SD)	51.57 (10.08)		46.8 (10.7)	
20–29	35 (1.7)		25 (5.0)	
30–39	230 (11.5)		105 (21.1)	
40–49	547 (27.4)		169 (33.9)	
50–59	691 (34.5)		123 (24.7)	
60–69	497 (24.8)		76 (15.3)	
Occupation				
Business owner	107 (5.4)		25 (5.0)	
Managing post	314 (15.7)		73 (14.7)	
Clerical work	254 (12.7)		83 (16.6)	
Technical job	254 (12.7)		67 (13.5)	
Sales staff	146 (7.3)		45 (9.0)	
Factory and labor work	159 (8.0)		38 (7.6)	
Service industry	165 (8.3)		46 (9.2)	
Educators and healthcare workers	90 (4.5)		30 (6.0)	
Self-employed business	86 (4.3)		26 (5.2)	
Commerce and industry self-employed	88 (4.4)		12 (2.4)	
Student	1 (0.1)		1 (0.0)	
Full-time homemaker	58 (2.9)		13 (2.6)	
Unemployment/age-limit retirement	213 (10.7)		24 (4.8)	
Agriculture, Forestry, or Fishery worker	8 (0.4)		4 (0.8)	
Caregiver for family/Under medical treatment	15 (0.8)		3 (0.1)	
Other	42 (2.1)		8 (1.6)	
Education				
Elementary school/junior high school	54 (2.7)		11 (2.2)	
High school (equivalent test)	615 (30.8)		138 (27.7)	
Two-year and career college	318 (15.9)		87 (17.5)	
Four-year college	927 (46.4)		237 (47.6)	
Graduate school	85 (4.3)		25 (5.0)	
Other	1 (0.1)			
Income (a year)				
< 1,000,000 JPY	179	(9.0)	35	(7.0)
1,000,000–1,999,999	114	(5.7)	28	(5.6)
2,000,000–2,999,999	223	(11.2)	60	(12.0)
3,000,000–3,999,999	271	(13.6)	70	(14.1)
4,000,000–4,999,999	269	(13.5)	65	(13.1)
5,000,000–5,999,999	263	(13.2)	81	(16.3)
6,000,000–6,999,999	192	(9.6)	50	(10.0)
7,000,000–7,999,999	138	(6.9)	35	(7.0)
8,000,000–8,999,999	108	(5.4)	25	(5.0)
9,000,000–9,999,999	73	(3.7)	18	(3.6)
10,000,000–15,000,000	128	(6.4)	22	(4.4)
15,000,000–	42	(2.1)	9	(1.8)
Allowance (a month)				
< 10,000	134	(6.7)	28	(5.6)
10,000–19,999	118	(5.9)	22	(4.4)
20,000–29,999	246	(12.3)	63	(12.7)
30,000–39,999	410	(20.5)	100	(20.1)
40,000–49,999	128	(6.4)	34	(6.8)
50,000–59,999	411	(20.6)	113	(22.7)
60,000–69,999	54	(2.7)	17	(3.4)
70,000–79,999	51	(2.6)	14	(2.8)
80,000–89,999	57	(2.9)	15	(3.0)
90,000–99,999	18	(0.9)	6	(1.2)
100,000–150,000	211	(10.6)	48	(9.6)
150,000–	162	(8.1)	38	(7.6)
PGSI				
Non–problem	512	(25.6)	75	(15.1)
Low–risk	416	(20.8)	67	(13.5)
Moderate-risk	650	(32.5)	158	(31.7)
Problem	422	(21.1)	198	(39.8)
Marital status				
Married	1,277 (63.9)		296 (59.4)	
Experience of divorce and/or bereavement	171 (8.6)		43 (8.6)	
Single	552 (27.6)		159 (31.9)	

### Gambling activities

Table 2 summarizes the types of gambling activities reported by participants (*N* = 2,000). Horse racing was the most common (75.1%), followed by lotteries (63.0%), pachinko (59.1%), and pachislot (45.0%). In contrast, only 8.8% reported experience with casino gambling, which is likely attributable to the legal prohibition of casinos in Japan. The frequency distributions also suggested that participation patterns differed across gambling activities, with some activities showing relatively higher proportions of frequent participation and others skewed toward lower frequencies (Table 2).

**Table 2:** Types and participation frequency of gambling activities among participants (*N* = 2,000).

	*n*	Never	Less than once a month	Once or more per month	Once or more per week
Horse racing	2000 100.0	498 24.9	279 14.0	513 25.7	710 35.5
Bicycle racing	2000 100.0	1,498 74.9	270 13.5	128 6.4	104 5.2
Boat racing	2000 100.0	1,447 72.4	286 14.3	150 7.5	117 5.9
Auto racing	2000 100.0	1719 86.0	164 8.2	76 3.8	41 2.1
Pachislot	2000 100.0	1,100 55.0	312 15.6	301 15.1	287 14.4
Pachinko	2000 100.0	818 40.9	327 16.4	455 22.8	400 20.0
Casino	2000 100.0	1824 91.2	140 7.0	22 1.1	14 0.7
Lottery	2000 100.0	739 37.0	752 37.6	222 11.1	287 14.4
Sports lottery	2000 100.0	1,305 65.3	393 19.7	144 7.2	158 7.9

### Prevalence of noriuchi and associations with gambling indicators

Among the 2,000 adult gamblers, 498 participants reported noriuchi experience in the past 12 months (433 men and 65 women; mean age = 46.8 years, SD = 10.7), representing 24.9% of the sample. Among them, 198 (39.76%) were classified as problem gamblers based on the Japanese PGSI (Table 3).

**Table 3:** Noriuchi participation frequency by gambling activity among participants who engaged in each gambling activity during the past 12 months.

	n	Never	Less than once a month	Once or more per month	Once or more per week
Horse racing	1,502 100.0	1,250 83.2	159 10.6	46 3.1	47 3.1
Bicycle racing	502 100.0	399 79.5	68 13.5	24 4.8	11 2.2
Boat racing	553 100.0	428 77.4	86 15.6	27 4.9	12 2.2
Auto racing	281 100.0	205 73.0	49 17.4	23 8.2	4 1.4
Pachislot	900 100.0	661 73.4	143 15.9	56 6.2	40 4.4
Pachinko	1,182 100.0	878 74.3	159 13.5	90 7.6	55 4.7
Casino	176 100.0	117 66.5	45 25.6	13 7.4	1 0.6
Lottery	1,261 100.0	1,060 84.1	142 11.3	35 2.8	24 1.9
Sports lottery	695 100.0	587 84.5	70 10.1	27 3.9	11 1.6

Table 4 presents the results of the ordinal logistic regression analyses for PGSI severity.

**Table 4:** Ordinal logistic regression analyses of PGSI severity.

Gambling activity	Noriuchi frequency	Adjusted OR (95% CI)	*p*
Horse racing	Less than monthly	3.72 (2.68–5.16)	3.553E-15
	Monthly	3.31 (1.88–5.83)	0.0000332
	Weekly	1.97 (1.14–3.39)	0.01462
Bicycle racing	Less than monthly	4.52 (2.57–7.92)	1.451E-07
	Monthly	8.69 (3.33–22.62)	9.615E-06
	Weekly	6.78 (1.78–25.88)	0.00508
Boat racing	Less than monthly	4.00 (2.48–6.47)	1.415E-08
	Monthly	15.87 (5.26–47.87)	9.289E-07
	Weekly	5.64 (1.66–19.18)	0.005595
Pachislot	Less than monthly	2.42 (1.70–3.44)	8.19E-07
	Monthly	3.42 (1.99–5.88)	8.732E-06
	Weekly	4.13 (2.15–7.95)	2.152E-05
Pachinko	Less than monthly	2.50 (1.78–3.50)	1.072E-07
	Monthly	3.16 (2.07–4.80)	8.336E-08
	Weekly	2.35 (1.39–3.98)	0.001485
Lottery	Less than monthly	2.75 (1.96–3.87)	5.836E-09
	Monthly	3.91 (2.02–7.58)	5.253E-05
	Weekly	1.40 (0.64–3.09)	0.403
Sports lottery	Less than monthly	4.36 (2.63–7.25)	1.266E-08
	Monthly	4.05 (1.94–8.45)	0.0001898
	Weekly	0.77 (0.22–2.65)	0.6775

Auto racing and casino gambling were excluded from the ordinal logistic regression analyses because sparse or zero cells in the higher-frequency noriuchi categories precluded stable model estimation.

### PGSI

The ordinal logistic regression analyses showed that less-than-monthly and monthly-or-more noriuchi participation were significantly associated with higher odds of belonging to a more severe PGSI category compared with no noriuchi participation for all gambling activities included in these analyses. In contrast, the association for weekly noriuchi participation differed across gambling activities. Weekly noriuchi participation was significantly associated with higher odds of more severe PGSI categories for pachislot, bicycle racing, boat racing, horse racing, and pachinko, whereas no significant associations were observed for lottery or sports lottery (toto).

Table 5 summarizes the results of the negative binomial regression analyses for monthly gambling days.

**Table 5:** Negative binomial regression analyses of monthly gambling days.

Gambling activity	Noriuchi frequency	IRR (95% CI)	*p*
Horse racing	Less than monthly	1.01 (0.88–1.16)	0.899
	Monthly	0.91 (0.72–1.16)	0.44
	Weekly	1.44 (1.15–1.81)	0.00179
Bicycle racing	Less than monthly	0.82 (0.67–1.02)	0.0627
	Monthly	1.28 (0.93–1.78)	0.13
	Weekly	1.29 (0.84–2.08)	0.272
Boat racing	Less than monthly	0.91 (0.76–1.1)	0.323
	Monthly	1.19 (0.89–1.6)	0.244
	Weekly	1.62 (1.09–2.49)	0.0216
Auto racing	Less than monthly	0.88 (0.69–1.13)	0.314
	Monthly	1.04 (0.74–1.48)	0.84
	Weekly	1.8 (0.93–3.92)	0.107
Pachislot	Less than monthly	1 (0.86–1.17)	0.958
	Monthly	1 (0.8–1.27)	0.992
	Weekly	1.64 (1.28–2.12)	0.000146
Pachinko	Less than monthly	1.03 (0.89–1.19)	0.69
	Monthly	0.94 (0.79–1.13)	0.518
	Weekly	1.75 (1.41–2.17)	3.65E-07
Casino	Less than monthly	1.06 (0.81–1.38)	0.676
	Monthly	1.16 (0.75–1.84)	0.505
	Weekly	1.62 (0.47–9.01)	0.5
Lottery	Less than monthly	0.94 (0.81–1.09)	0.377
	Monthly	1.27 (0.97–1.67)	0.0866
	Weekly	1.34 (0.99–1.87)	0.0701
Sports lottery	Less than monthly	0.84 (0.68–1.05)	0.115
	Monthly	1.11 (0.82–1.53)	0.498
	Weekly	1.42 (0.91–2.33)	0.141

### Monthly gambling days

Negative binomial regression analyses indicated that less than monthly and monthly or more noriuchi participation were not significantly associated with the number of monthly gambling days for any gambling activity. However, the association for weekly noriuchi participation differed across gambling activities. Compared with participants who did not engage in noriuchi, weekly noriuchi participation was significantly associated with a greater number of monthly gambling days for horse racing, boat racing, pachislot, and pachinko. No significant associations were observed for bicycle racing, auto racing, casino gambling, lottery, or sports lottery (toto).

Table 6 presents the results of the multiple linear regression analyses for log-transformed monthly gambling expenditure.

**Table 6:** Multiple linear regression analyses of log-transformed monthly gambling expenditure.

Gambling activity	Noriuchi frequency	β (95% CI)	*p*
Horse racing	Less than monthly	−0.07 (−0.358–0.219)	0.636
	Monthly	0.139 (−0.36–0.638)	0.586
	Weekly	0.341 (−0.153–0.834)	0.176
Bicycle racing	Less than monthly	−0.612 (−1.078–0.145)	0.0103
	Monthly	0.215 (−0.514–0.945)	0.563
	Weekly	−0.113 (−1.162–0.936)	0.833
Boat racing	Less than monthly	−0.296 (−0.733–0.141)	0.184
	Monthly	0.172 (−0.541–0.885)	0.636
	Weekly	0.513 (−0.522–1.548)	0.331
Auto racing	Less than monthly	−0.504 (−1.036–0.027)	0.0629
	Monthly	0.023 (−0.733–0.78)	0.951
	Weekly	0.522 (−1.109–2.154)	0.529
Pachislot	Less than monthly	0.065 (−0.26–0.39)	0.694
	Monthly	0.02 (−0.466–0.506)	0.936
	Weekly	0.923 (0.36–1.485)	0.00133
Pachinko	Less than monthly	−0.118 (−0.432–0.197)	0.463
	Monthly	−0.043 (−0.436–0.349)	0.828
	Weekly	0.556 (0.069–1.042)	0.0252
Casino	Less than monthly	−0.199 (−0.839–0.44)	0.54
	Monthly	0.194 (−0.882–1.271)	0.722
	Weekly	1.255 (−2.333–4.843)	0.491
Lottery	Less than monthly	−0.296 (−0.609–0.017)	0.0638
	Monthly	−0.039 (−0.634–0.556)	0.898
	Weekly	0.125 (−0.589–0.84)	0.731
Sports lottery	Less than monthly	−0.566 (−0.998–0.133)	0.0104
	Monthly	0.178 (−0.473–0.83)	0.591
	Weekly	−0.331 (−1.337–0.674)	0.518

### Monthly gambling expenditure

Multiple linear regression analyses using log-transformed monthly gambling expenditure showed that less than monthly and monthly or more noriuchi participation were not significantly associated with monthly gambling expenditure in most gambling activities compared with no noriuchi participation. However, less than monthly noriuchi participation was significantly associated with lower monthly gambling expenditure for bicycle racing and sports lottery (toto). The association for weekly noriuchi participation differed across gambling activities. Weekly noriuchi participation was significantly associated with higher monthly gambling expenditure for pachislot and pachinko, whereas no significant associations were observed for horse racing, bicycle racing, boat racing, auto racing, casino gambling, lottery, or sports lottery (toto).

## Discussion

This study aimed to clarify the prevalence of noriuchi among 2,000 Japanese adult gamblers and to explore how noriuchi participation frequency relates to gambling involvement (monthly gambling days and expenditure) and problem gambling severity (PGSI).

The primary finding was that approximately one quarter of the sampled adult gamblers (24.9%) reported engaging in noriuchi during the past year. Although direct benchmarks for determining whether this proportion is relatively high or low in the broader context of gambling behavior in Japan are limited, the finding indicates that noriuchi is practiced by a substantial minority of gamblers.

Weekly noriuchi participation, but not less-than-monthly or monthly-or-more participation, was associated with a greater number of monthly gambling days for several gambling activities. This finding suggests that frequent engagement in noriuchi may be associated with repeated gambling participation rather than occasional involvement. This interpretation is consistent with previous studies on social facilitation, which suggest that gambling in the presence of others can increase gambling engagement (Rockloff and Dyer, 2007; Rockloff et al., 2011) and prolong gambling sessions among friends (Griffiths and Parke, 2003). Because this was a cross-sectional survey, causal mechanisms cannot be determined. Nevertheless, one plausible interpretation is that gambling together may increase opportunities to gamble, leading to more days of participation. Given that noriuchi presupposes multiple participants, invitations and accompaniment by acquaintances may increase participation opportunities. This interpretation aligns with studies suggesting that peers and the social environment can influence gambling initiation and engagement (Parrado-González et al., 2023; Donati et al., 2023).

In contrast, noriuchi participation showed little consistent association with monthly gambling expenditure. Weekly noriuchi participation was associated with higher gambling expenditure only for pachislot and pachinko, whereas no significant associations were observed for most other gambling activities. These findings suggest that noriuchi does not necessarily increase individual gambling expenditure across gambling activities. One possible explanation is that noriuchi involves pooling gambling funds among participants (e.g., two people jointly wagering 30,000 JPY implies 15,000 JPY per person), which could reduce the amount contributed by each individual even when gambling participation becomes more frequent. Consequently, noriuchi may be associated with greater gambling involvement without a proportional increase in individual financial expenditure. However, expenditure is self-reported and highly variable, and this study did not assess detailed within-session decision processes (e.g., how groups negotiate continuation). Future studies could use more precise expenditure recording and assess how pooling arrangements and in-session coordination influence spending dynamics.

These findings suggest that the association between noriuchi and problem gambling severity may not be explained solely by differences in gambling frequency or expenditure. Other processes associated with gambling in a group setting may also be relevant; however, these processes were not directly assessed in the present study.

Several limitations should be noted. First, the cross-sectional online design precludes conclusions about causal direction (e.g., whether noriuchi increases gambling involvement, or whether individuals who already gamble more are more likely to engage in noriuchi). Longitudinal designs that track changes in accompaniment and stake-sharing over time are needed. Second, all measures were self-reported and may be affected by recall error, ambiguous reporting, and social desirability bias. Such measurement error may be particularly large for expenditure and frequency and could have reduced statistical power to detect group differences. Third, participants were screened to include individuals who gambled at least monthly in the past year; therefore, generalizability to less frequent gamblers or broader populations may be limited. Future work should replicate these findings in broader samples and distinguish variations in noriuchi implementation (e.g., relationship to co-gamblers, settlement rules, and whether explicit agreements are made).

## Conclusion

Noriuchi was reported by roughly one in four Japanese adult gamblers in this sample. After adjusting for demographic covariates, noriuchi participation was associated with greater problem gambling severity across several gambling activities. In contrast, associations with monthly gambling days and expenditure were more limited and varied across gambling activities, with significant increases primarily observed among those engaging in noriuchi weekly for certain gambling activities. These findings suggest that noriuchi is associated with problem gambling severity, whereas its associations with gambling frequency and individual gambling expenditure are less consistent.

## Data Availability

The original contributions presented in the study are included in the article/supplementary material, further inquiries can be directed to the corresponding author.
